# Cytokines help suggest aplastic anemia with pulmonary bacterial or co-fungal infection

**DOI:** 10.1038/s41598-022-22503-7

**Published:** 2022-11-01

**Authors:** Jinping Zhang, Zefeng Yang, Peng Hu, Xin Guan, Chaoran Zhang, Yunlian Zou, Huiyuan Li, Tonghua Yang, Yue Cao, Renbin Zhao, Zengzheng Li

**Affiliations:** 1 Department of Hematology, The First People’s Hospital of Yunnan Province, Affiliated Hospital of Kunming University of Science and Technology, 157 Jinbi Road, Kunming, 650032 China; 2 Yunnan Blood Disease Clinical Medical Center, The First People’s Hospital of Yunnan Province, Kunming, China; 3 Yunnan Blood Disease Hospital, The First People’s Hospital of Yunnan Province, Kunming, China; 4 National Key Clinical Specialty of Hematology, The First People’s Hospital of Yunnan Province, Kunming, China; 5 Yunnan Province Clinical Research Center for Hematologic Disease, Kunming, China; 6 Emergency Department of Yunnan Provincial Hospital of Traditional Chinese Medicine, Affiliated to Yunnan University of Traditional Chinese Medicine, Kunming, China

**Keywords:** Biomarkers, Diseases, Medical research

## Abstract

Although aplastic anemia (AA) does not come under the category of blood malignant diseases, the infection that frequently occurs in this bone marrow failure can make it worse. Pulmonary infection is the most prevalent but limiting clinical diagnosis. To find biomarkers predicting bacterial or bacterial-combined fungal infections in the lungs, we reviewed 287 AA medical records including 151 without any infection, 87 with pure pulmonary bacterial infection, and 49 with bacterial and fungal infection were reviewed. There were substantial changes in IL-17F, IL-17A, IFN-γ, IL-6, IL-8, and IL-10 levels between the non-infected and lung bacterial infection groups (P < 0.05). Further, a significant variation in IL-17A, TNF-β, IL-1β, IL-2, IL-4, IL-6, IL-8, IL-10, IL-22, and IL-12p70, between the uninfected group and the pulmonary bacterial and fungal infection group (P < 0.05) was observed. The results further revealed significant differences in TNF-β, IL-12p70, IL-6, IL-8, and IL-10 between the pulmonary bacterial infection group and the fungal infection group (P < 0.05). Moreover, by calculating ROC and cut-off values, we determined that IL-6 (AUC = 0.98, Cut-off = 14.28 pg/ml, P = 0.0000) had a significant advantage than other cytokines, body temperature (AUC = 0.61, P = 0.0050), PCT (AUC = 0.57, P = 0.0592), and CRP (AUC = 0.60, P = 0.0147) in the detection of lungs bacterial infections. In addition, IL-6 (AUC = 1.00, Cut-off = 51.50 pg/ml, P = 0.000) and IL-8 (AUC = 0.87, Cut-off = 60.53 pg/ml, P = 0.0000) showed stronger advantages than other cytokines, body temperature (AUC = 0.60, P = 0.0324), PCT (AUC = 0.72, Cut-off = 0.63 ng/ml, P = 0.0000) and CRP (AUC = 0.79, Cut-off = 5.79 mg/l, P = 0.0000) in distinguishing bacteria from fungi. This may suggest that IL-8 may play a role in differentiating co-infected bacteria and fungi. Such advantages are repeated in severe aplastic anemia (SAA) and very severe aplastic anemia (VSAA).In conclusion, aberrant IL-6 elevations in AA patients may predict the likelihood of bacterial lung infection. The concurrent increase of IL-6 and IL-8, on the other hand, should signal bacterial and fungal infections in patients.These findings may help to suggest bacterial or fungal co-infection in patients with AA (Focus on VSAA and SAA).

## Introduction

“Aplastic” refers to the inability of bone marrow to form blood, the end-organ effect of diverse pathophysiologic mechanisms. Mostly, Aplastic anemia (AA) patients have pancytopenia, with decreased levels of platelets and white blood cells^1,2^. The immune function of AA patients is always low, which makes AA patients susceptible to infection, and infection is also a significant cause of death in AA patients^3,4^. Infection, especially early pulmonary infection, has always been a fundamental problem for hematologists, mainly because pulmonary infection, especially early infection, is not easy to diagnose. Body temperature, PCT, and CRP were less-sensitive in multiple infection studies on hematological malignancies^5,6^. The main problem with commonly used sputum culture is the low positive rate ^7^.Computerized tomography (CT) is still the most important tool for lung infection clinical diagnosis. However, it may not well diagnose the disease at the early stages, and may be restricted by the health center.

None of the laboratory tests for lung infections are perfect. In addition to the above examinations, lung lavage fluid culture and lung lavage fluid microbial next-generation sequencing(mNGS) have a wider and more accurate identification of pathogenic microorganisms in basic research and clinical experiments, and are also subject to many restrictions in clinical use (such as price, long sampling process and time to obtain results)^8^, especially for some blood system diseases. Like most developing countries or economically backward regions, the biggest problem we have is that because of economic reasons, the lung lavage fluid culture and lung lavage fluid micro next generation sequencing (mNGS), especially mNGS, can only be developed among a small number of people. Therefore, more auxiliary examinations are needed to strengthen the identification of pulmonary infection.

The limitations of these common examination items can easily lead to early lung infection not being detected in time, which further develops into serious infection, affecting the treatment of patients, and even affecting the survival of patients. In other words, the initial pulmonary infection is often found and treated after the infection is aggravated. Therefore, it is important to provide evidence of infection, which may help to accelerate the diagnosis of pulmonary infection.Cytokines are now gaining more attention, and previous studies have found cytokines to be more sensitive to lung infection than PCT, CRP, and body temperature in the absence of other diseases^9,10^. Furthermore, cytokines have the advantages of requiring less quantity of samples for detection, eliminating the need to consider secondary pollution, and allowing for faster flow cytometry detection. Although cytokines have been shown to have a strong relationship with inflammation, they have also been used to predict inflammation.

Our team has also been working on studying cytokines indicating pulmonary infection in patients with hematological diseases. Previous research results suggest that cytokines predicting pulmonary infection also apply to hematological tumors. But we also found differences in cytokine expression between different types of blood diseases^5,11^: considering the difference in disease, especially the difference between non-tumor disease and tumor disease. The primary focus of the current study is to investigate AA patients(Focus on VSAA and SAA) and explore the expression pattern of cytokines in the lungs of AA patients with bacterial infection and find the best potential biomarkers.

## Material and methods

### Declaration

All methods were performed in accordance with the relevant guidelines and regulations.The “Declaration of Helsinki” and the “International Code of Ethics for Human Biomedical Research” jointly formulated by the International Council of Medical Science Organizations and the World Health Organization were strictly followed throughout this study. At the same time, the project was approved by the Ethics Committee of the First People's Hospital of Yunnan Province, number: KHLL2022-KY075. All study participants provided informed consent simultaneously.

### Study participants and data collection

A total of 287 non-children AA medical records from patients who visited Yunnan First People’s Hospital’s Department of Hematology from June 2017 to May 2022 were evaluated for this study. It consists of 70 VSAA cases and 217 SAA cases. At the initial examination of the visit factor, we recorded the body temperature, Th1/Th2/Th17, CRP, and PCT cytokines. Refer to the following diagnostic criteria for bacterial lung infection^12–14^; bacterial lung infection, suspected or confirmed, irrespective of the presence or absence of bacteria in respiratory specimens, and is classified based on clinical symptoms and diagnostic standards. Cough that is becoming worse or new, shortness of breath, hypoxia, or dyspnea are all clinical criteria. Chest X-rays to detect inflammatory changes and sputum culture to detect bacterial infection or other auxiliary tests. In certain patients with suspected pulmonary disease, chest CT scans were used to confirm bacterial infection. For patients with bacterial and fungal infection, we only included patients with positive bronchoalveolar lavage fluid (BAL)1,3- β- D glucan test (G test) or/and galactomannan test (GM test) culture.

### Statistical analysis

The data were initially examined for normality to see whether they have a normal distribution. Then, a *t* test or Mann–Whitney *U* test was conducted on independent samples. Continuous data are represented by the median and interquartile range (IQR), whereas percentages and frequencies describe categorical variables. We calculated the area under the curve (AUC) using the ROC curve to evaluate the diagnostic value of each parameter. Using the highest Youden index, the optimal Cut-off was determined. The GraphPad Prism 7 software was utilized to generate distribution plots, and the IBM SPSS 21.0 (Armonk, NY, USA) was used for statistical analysis. P-values of < 0.05 on (two-sided) were considered statistically significant.

### Determination of cytokines

The kit used to detect serum cytokines is Aimplex Cytokine (QuantoBio, Tianjin, China) with a detection range of 1–2500 pg/ml, and the detected cytokines include Th (1/2/17), namely: interferon (IFN) -γ, IL -(1β, 2, 4, 5, 6, 8, 10, 12p70, 17A, 17F, 22) and tumor necrosis factor (TNF-α and β) using flow cytometry (NovoCyte D3000).

## Results

### Patients characteristics

**Figure 1:**
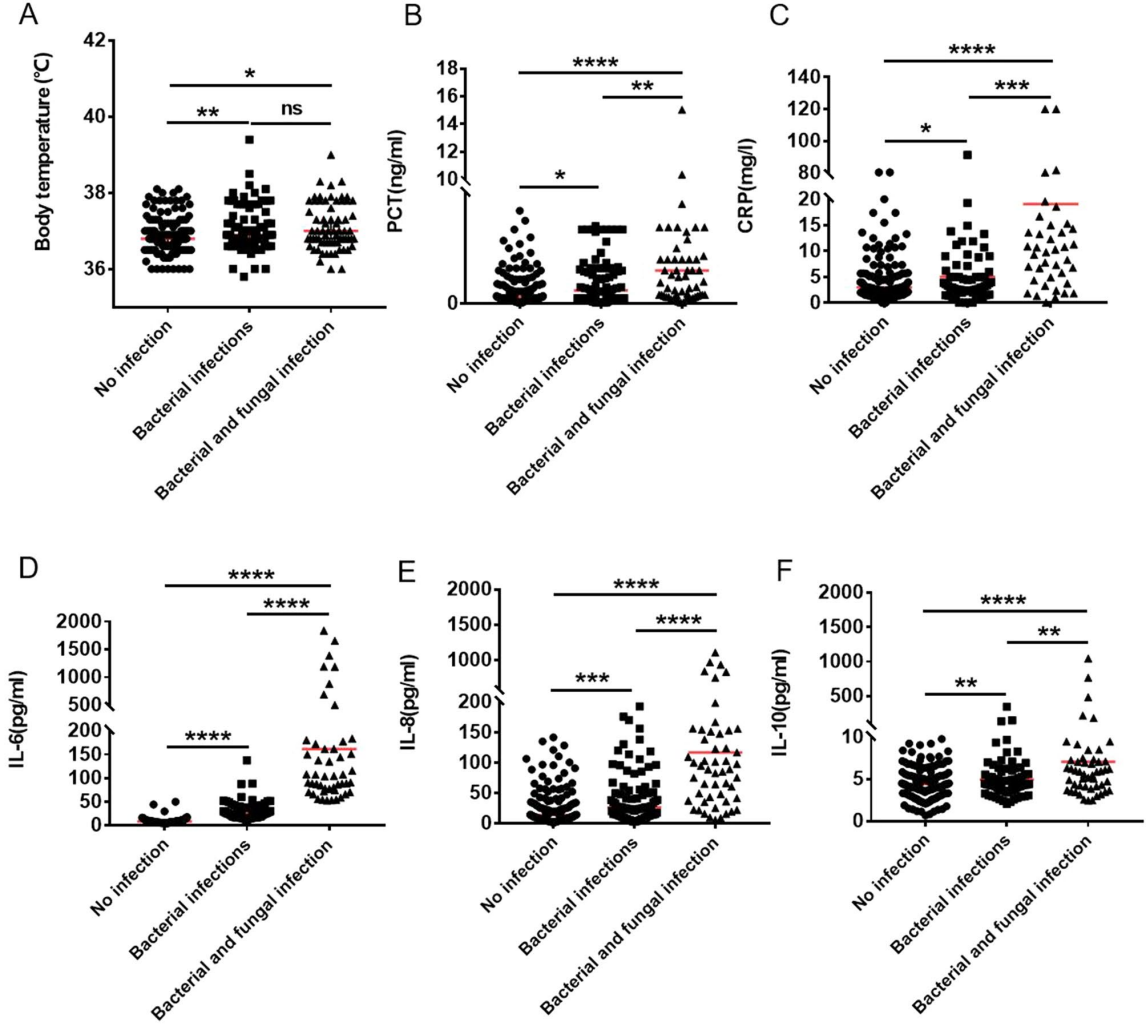
The Mann–Whitney *U* test was used to test the differences among patients with respiratory tract bacterial infection, patients with respiratory tract bacterial complicated with fungal infection and patients with no bacterial infection. (**A**)Body temperature, (**B**)PCT, (**C**)CRP, (**D**) IL-6, (**E**) IL-8, (**F**) IL-10. *P < 0.05, **P < 0.01, ***P < 0.001, ****P < 0.0001, *ns* no statistical difference.

1.4/5Among the 287 medical records, there were 125 females and 162 males, the median age was 29 (22–49) years, 151 patients without any infection, the median body temperature was 36.8 (36.6–37.0) °C (the mean value is 36.86 ± 0.46 °C), and the median PCT was 0.06 (0.04–0.2) ng/ml, median CRP 3.00 (1.55–8.78). Eighty-seven patients had a simple pulmonary bacterial infection, the median body temperature was 36.90 (36.70–37.60) °C(the mean value is 37.07 ± 0.60 °C), the median PCT was 0.12 (0.04–0.32) ng/ml, and the median CRP was 5.00 (2.48–29.00). Forty-nine patients had bacterial and fungal infections, with a median body temperature of 36.8 (36.7–37.6) °C (the mean value is 37.08 ± 0.63 °C), median PCT of 0.26 (0.08–1.10) ng/ml, and median CRP of 19.10 (10.04–39.15) (Table 1 and Fig. 1A–C).

**Table 1:** Patient characteristics.

	A: no infection (n = 151)	B: bacterial infection of the lungs (n = 87)	C: bacterial and fungal infection of the lungs (n = 49)	P (A VS B	P (A VS C)	P (B VS C)
Gender (male/female)	74/77	53/34	35/14	–	–	–
Age (years old)	35.00 (23–52)	26.00 (20–52)	25.00 (25–42)	0.1322	0.3976	0.0076
Body temperature (°C)	36.8 (36.6–37.0)	36.9 (36.70–37.60)	36.8 (36.7–37.6)	0.0048	0.0324	0.7677
PCT(ng/ml)	0.06 (0.04–0.2)	0.12 (0.04–0.32)	0.26 (0.08–1.10)	0.0462	0.0000	0.0006
CRP (mg/l)	3.00 (1.55–8.78)	5.00 (2.48–29.00)	19.10 (10.04–39.15)	0.0147	0.0000	0.0003

The difference in the expression of Age, body temperature, PCT and CRP between the patient’s lungs with bacterial infection and without infection was detected by the Mann–Whitney *U* test.

P < 0.05 means there is a statistical difference.

### Cytokine expression

In our study cohort IL-4, IL-5, IL-6, IL-8, IL-10, IL-12p70, IL-1β, IL-2, IFN-γ, TNF-α, TNF-β, IL-17A, IL-17F and IL-22 The median expression levels in non-infected patients were 2.72 (1.82–3.95) pg/ml, 2.15 (1.59–2.98) pg/ml, 8.19 (6.10–10.41) pg/ml, 14.13 (6.75–35.46) pg/ml, 4.38 (3.3–6.44) pg/ml, 3.94 (3.27–5.71) pg/ml, 1.64 (1.16–2.16) pg/ml, 3.08 (2.23–4.80) pg/ml, 2.87 (1.89–3.82) pg/ml, 2.84 (2.02–3.93) pg/ml, 3.16 (2.57–3.90) pg/ml, 2.29 (1.37–3.33) pg/ml, 3.36 (2.47–4.67) pg/ml, 1.01 (0.58–1.80) pg/ml (Table 2).

**Table 2:** Cytokine expression.

Parameter	A: no infection	B: bacterial infection	C: fungal and bacterial infection	P (A VS B)	P (A VS C)	P (B VS C)
IL-4 (pg/ml)	2.72 (1.82–3.95)	2.84 (1.80–5.00)	3.55 (2.08–6.67)	0.3332	0.0170	0.1406
IL-5 (pg/ml)	2.15 (1.59–2.98)	2.29 (1.71–3.64)	2.19 (1.45–3.71)	0.1389	0.8949	0.4078
IL-6 (pg/ml)	8.19 (6.10–10.41)	26.43 (17.40–37.48)	104.73 (75.80–260.86)	0.0000	0.0000	0.0000
IL-8 (pg/ml)	14.13 (6.75–35.46)	26.62 (14.19–60.58)	103.15 (46.06–209.31)	0.0001	0.0000	0.0000
IL-10 (pg/ml)	4.38 (3.3–6.44)	4.96 (3.95–8.20)	7.08 (4.18–15.61)	0.0062	0.0000	0.0075
IL-12p70 (pg/ml)	3.94 (3.27–5.71)	4.29 (3.44–6.52)	5.80 (4.32–6.83)	0.0741	0.0196	0.0167
IL-1β (pg/ml)	1.64 (1.16–2.16)	1.75 (1.41–2.32)	1.93 (1.38–2.45)	0.1343	0.0001	0.1668
IL-2 (pg/ml)	3.08 (2.23–4.80)	3.49 (2.60–5.28)	4.44 (3.04–6.03)	0.0910	0.0087	0.0949
IFN-γ (pg/ml)	2.87 (1.89–3.82)	3.07 (2.43–4.10)	3.56 (2.10–4.56)	0.0244	0.0742	0.8305
TNF-α (pg/ml)	2.84 (2.02–3.93)	3.07 (2.05–4.42)	3.17 (2.42–4.36)	0.4348	0.1165	0.2170
TNF-β (pg/ml)	3.16 (2.57–3.90)	3.20 (2.66–3.75)	3.73 (2.99–4.09)	0.8191	0.0258	0.0324
IL-17A (pg/ml)	2.29 (1.37–3.33)	2.69 (1.59–4.23)	3.40 (1.93–4.69)	0.0321	0.0023	0.1358
IL-17F (pg/ml)	3.36 (2.47–4.67)	4.23 (13.06–6.50)	3.99 (2.48–5.69)	0.0005	0.0642	0.2383
IL-22 (pg/ml)	1.01 (0.58–1.80)	1.24 (0.62–2.59)	1.39 (0.92–1.98)	0.1850	0.0392	0.1514

The difference in the expression of cytokines between the patient’s lungs with bacterial infection and without infection was detected by the Mann–Whitney *U* test.

P < 0.05 means there is a statistical difference.

The median expression levels in patients with pulmonary bacterial infections were 2.84 (1.80–5.00) pg/ml, 2.29 (1.71–3.64) pg/ml, 26.43 (17.40–37.48) pg/ml, 26.62 (14.19–60.58) pg/ml, 4.96 (3.95–8.20) pg/ml, 4.29 (3.44–6.52) pg/ml, 1.75 (1.41–2.32) pg/ml, 3.49 (2.60–5.28) pg/ml, 3.07 (2.43–4.10) pg/ml, 3.07 (2.05–4.42) pg/ml, 3.20 (2.66–3.75) pg/ml, 2.69 (1.59–4.23) pg/ml, 4.23 (13.06–6.50) pg/ml, 1.24 (0.62–2.59) pg/ml. The median expression levels in patients with pulmonary comorbid bacteria and fungi were 3.55 (2.08–6.67) pg/ml, 2.19 (1.45–3.71) pg/ml, 104.73 (75.80–260.86) pg/ml, 103.15 (46.06–209.31) pg/ml, 7.08 (4.18–15.61) pg/ml, 5.80 (4.32–6.83) pg/ml, 1.93 (1.38–2.45) pg/ml, 4.44 (3.04–6.03) pg/ml, 3.56 (2.10–4.56) pg/ml, 3.17 (2.42–4.36) pg/ml, 3.73 (2.99–4.09) pg/ml, 3.40 (1.93–4.69) pg/ml, 3.99 (2.48–5.69) pg/ml, 1.39 (0.92–1.98) pg/ml (Table 2). Among the three groups of patients, there were significant differences in IL-6, IL-8, IL-10, IFN-γ, IL-17A, and IL-17F between the non-infection group and the pulmonary bacterial infection group (P < 0.05) as given in Table 2, Fig. 1D–F, and Supplementary Fig. 1D–F.

There were significant differences in IL-4, IL-6, IL-8, IL-10, IL-12p70, IL-1β, IL-2, TNF-β, IL-17, and IL-22 between the uninfected group and the pulmonary bacterial and fungal infection group (P < 0.05) (Table 2, Fig. 1D–F, Supplementary Fig. 1A–E,G,H). There were significant differences in IL-6, IL-8, IL-10, IL-12p70, and TNF-β between the pulmonary bacterial infection group and the pulmonary bacterial, fungal infection group (P < 0.05) (Table 2, Fig. 1D–F, Supplementary Fig. 1A,G).

### Determination of specific indicators

**Figure 2:**
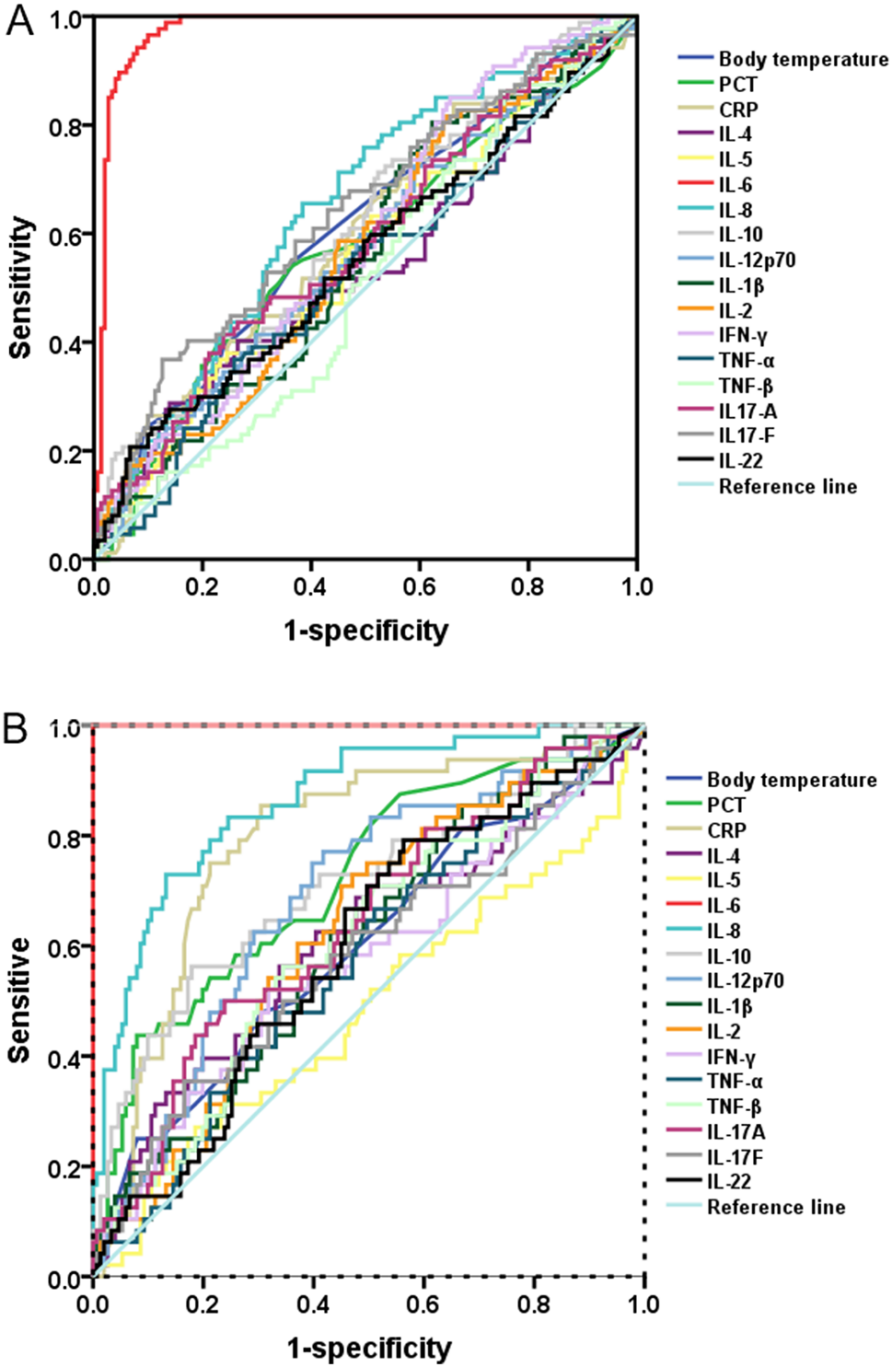
(**A**) the ROC curves of cytokine, PCT, Body temperature and CRP in patients with respiratory tract bacterial infection were compared with those without respiratory tract infection. (**B**) the ROC curves of cytokine, PCT, Body temperature and CRP in patients with respiratory tract bacterial and fungal infection compared with those without infection.

Cut-off values were calculated from ROC curves, retaining potentially valuable cytokines, and compared with body temperature, PCT, and CRP. Our results show that IL-6 (AUC = 0.98, Cut-off = 14.28 pg/ml, P = 0.0000) has better sensitivity than other cytokines, body temperature AUC = 0.61, P = 0.0050), PCT (AUC = 0.57, P = 0.0592) and CRP (AUC = 0.60, P = 0.0147) for predicting bacterial infection of the lung (Table 3 and Fig. 2A). Further, the AUC and Cut-off values between no infection and bacterial co-infection with fungal infection were further calculated. Surprisingly, IL-6 (AUC = 1.00, Cut-off = 51.50 pg/ml, P = 0.0000) also had a strong advantage, and IL-8 (AUC = 0.87, Cut-off = 60.53 pg/ml, P = 0.0000) also showed stronger than other cytokines, body temperature (AUC = 0.60, P = 0.0324, PCT (AUC = 0.72, Cut-off = 0.63 ng/ml, P = 0.0000) and CRP (AUC = 0.79, Cut-off = 5.79 mg/l, P = 0.0000) (Table 3 and Fig. 2B).

**Table 3:** Cut-off values of cytokines (AUC ≥ 70), CRP, PCT and body temperature between no infection and bacterial lung infection.

Parameter	Between no infection and bacterial lung infection		Cut-off	Between no infection and pulmonary bacterial and fungal infection		
	AUC	P		AUC	P	Cut-off
Body temperature (°C)	0.61	0.0050	–	0.60	0.0324	–
PCT (ng/ml)	0.57	0.0592	–	0.72	0.0000	0.63
CRP (mg/l)	0.60	0.0147	–	0.79	0.0000	5.79
IL-6 (pg/ml)	0.98	0.0000	14.18	1.00	0.0000	51.50
IL-8 (pg/ml)	0.65	0.0001	–	0.87	0.0000	60.53
IL-10 (pg/ml)	0.61	0.0062	–	0.71	0.0000	6.83

Calculate the Cut-off values of cytokines (AUC ≥ 70), CRP, PCT and body temperature by ROC curve.

P < 0.05 means there is a statistical difference.

The common clinical types of AA are severe aplastic anemia and severe aplastic anemia. In our study cohort, there are 70 VSAA patients and 217 SAA patients. By comparison, we found that only IL-8 had statistical difference in patients without infection (P = 0.0004), only IL-6 had statistical difference in patients with pulmonary bacterial infection (P = 0.0120), and IL-12P70 (P = 0.0046) and INF- γ (P = 0.0279) in patients with pulmonary bacterial and fungal infection had statistical difference (Supplementary Table 1). Furthermore, we calculated the AUC of cytokines in SAA and VSAA respectively by ROC curve. Our results show that the infection sensitive indicators in SAA and VSAA are similar, and such results are consistent with those in AA (VSAA + SAA) (Supplementary Table 2, Supplementary Fig. 2A–D and Table 3). This may indicate that both IL-6 and IL-8 can indicate the lung bacterial infection of VSAA/SAA and the co infection of lung bacteria and fungi.

## Discussion

Infection is prevalent in AA with bone marrow failure because immunosuppression is prevalent during the treatment of hematological diseases Among them, SAA and VSAA are the most frequently infected in clinic^15,16^. Due to the lack of corresponding clinical symptoms of early bacterial infection in the lungs, it is easy to be ignored or misdiagnosed at the time of diagnosis. This often leads to aggravation or even death of patients, so it is necessary to increase auxiliary diagnosis indicators. In this study, the abnormal expression of IL-6 was found suitable for the pulmonary bacterial infection of AA. When the expression level is high, it may indicate a pulmonary bacterial and fungal infection in AA patients. In addition, the abnormal expression of IL-8 may help distinguish whether AA patients develop a bacterial co-fungal infection, which is different from the previous report that the increase of cytokines contributes to the bacterial lung infection in hematological cancers. Simultaneous expression of IL-10, IL-8, and IL-6 is a decisive advantage in newly diagnosed hematological malignancies and pediatric patients^5,6,17^.

Consistent with lymphoma and other reports, IL-6 was the most dominant of all markers^11,18,19^; our findings provide insight into the availability of IL-6 in AA for predicting bacterial lung infection. Our results revealed that IL-10, IL-8, IL-6, IFN-, IL-17A, and IL-17F levels in patients with bacterial lung illness were abnormally higher than those without infection (Table 2). This is different from our study on non-Hodgkin's lymphoma, where only IL-6, IL-8, IL-10, and IFN-γ have intersection indicators, while non-Hodgkin’s lymphoma Abnormal TNF-β is also present^11^. These differences may be primarily due to the disease itself, e.g., TNF-β can be related to tumor burden^20^. In addition, TNF-β is also a non-Hodgkin’s poor prognostic factor^21^. Therefore, it is also necessary to study the cytokines of each blood system disease separately, which is beneficial to increase the reliability of the application of cytokines in different conditions.

Previous research has found that invasive pulmonary aspergillosis causes an increase in Th1/17 cytokines in individuals with hematological diseases and that IL-6 and IL-8 are strongly related to invasive pulmonary aspergillosis^22,23^. *Aspergillus fumigatus* protease induces IL-6 and IL-8 release from A549 lung epithelial cells and primary epithelial cells^24^.In addition, fungal spores also promote the secretion of IL-8 from A549 lung epithelial cells^25,26^.IL-6 expression plays a crucial role in protective immunity in mice infected with Aspergillus^27^. Further, the *Aspergillus flavus* can also induce the expression of IL-8^28^.

Furthermore, abnormally high expression of IL-6 and IL-8 may indicate poor mortality in individuals with hematological malignancies who have pneumococcal infection^29^. These data suggest that IL-6 and IL-8 are overexpressed in fungal-infected patients, and numerous hematological malignancies have been documented. Our results complement that IL-6 and IL-8 are similarly aberrant in AA expression in hematological non-neoplastic diseases and may have a role in discriminating bacterial or co-fungal infections because only IL-6 had a solid sensitivity to bacterial lung infection in our results. But after bacterial infection with a fungus, the IL-6 has a strong sensitivity. IL-8 also possesses better sensitivity than PCT, CRP, and body temperature. (Table 3, Fig. 2A,B). However, we analyzed SAA and VSAA separately, and the results were repeated, which may indicate that this model is applicable to both SAA and VSAA(Supplementary Table 2, Supplementary Fig. 2A–D and Table 3).

Our study is a retrospective study, and the biggest regret is that the number of patients with accurate identification of pathogenic bacteria is small and we did not include it in the analysis. For lung infection, the technology widely used to identify pathogenic bacteria in most countries and regions is still sputum culture, but the positive rate of sputum culture is only about 30%^7,30^. The more popular studies are obtaining samples directly from affected lesions by bronchoalveolar lavage (BAL) or by safe brushing with bronchoscopy followed by culture or mNGS, which is an invasive procedure^31^. For the invasive examination of bone marrow failure disease AA in the state of immune deficiency, especially VSAA. People often worry about increasing microbial infection and causing trauma. Also similar to sputum cultures, routine phenotypic diagnosis of BAL sample for pathogenic microorganism detection (including G test and GM test) usually takes 48 to 72 h, and the sensitivity range is only 30% to 60%, leading to undesirable delays in pathogen detection and initiation of antimicrobial therapy, potentially leading patients to miss optimal timing of therapy^32–34^. Especially when invasive aspergillosis occurs, the mortality may exceed 50%^35^.

The detection of microorganisms by mNGS through BAL is the most accurate and extensive at present, and it is also a detection project carried out in developed areas and comprehensive hospitals with perfect conditions. In addition to concerns about the sampling risk and the long testing process (About 2–3 weeks), the core challenge of mNGS is to distinguish pathogens from colonized microorganisms, which also complicates the interpretation of results and is not conducive to clinician interpretation^36,37^. In contrast to mGNS, although the presence of non-specific cytokines, the colonized flora generally does not lead to cytokine disturbance^38–40^. In addition, the inspection fee is relatively high, which must be considered in developing countries and regions. But it must be acknowledged that mNGS can identify microorganisms more widely and more accurately, which has high value for both clinical testing and scientific research.

our research is carried out in developing countries, which may also reflect the common problems in most economically backward regions. Although IL-6 is generally elevated in infected patients, we still hope to elucidate the expression of cytokines in pulmonary bacterial or/and fungal infections of AA patients, which may provide clues for clinical practice in AA.The most urgent task for us as clinicians is to detect the presence of infection as early as possible and to intervene as soon as possible, especially in bone marrow failure diseases such as AA. All in all, we still hope to find more sensitivity indicators from more commonly used clinical tests to help clinicians detect pulmonary bacterial or co-fungal infection in patients as early as possible.

## Conclusion

Cytokines are abnormally expressed in AA in pulmonary infection, and the abnormal increase in IL-6 suggests that the patient has a pulmonary bacterial infection. However, when IL-8 and IL-6 are elevated simultaneously, it may indicate that the patient has a bacterial and fungal infection. These findings may help suggest bacterial or concurrent bacterial and fungal infections in the lungs of SAA and VSAA patients.

## Supplementary Information

Supplementary Information 1.

Supplementary Information 2.

## Data Availability

All data generated or analysed during this study are included in this published article [and its supplementary information files].
