# Supplementary material for: Cytokines help suggest aplastic anemia with pulmonary bacterial or co-fungal infection
Source: Sci Rep. 2022 Nov 1;12:18373. doi: 10.1038/s41598-022-22503-7 (PMC9626605; doi:10.1038/s41598-022-22503-7)
Supplement: Supplementary file 1 — Supplementary Information 1. [file 41598_2022_22503_MOESM1_ESM.docx]

Supplementary Table1. Expression of cytokines between VSAA and SAA

|  | **Bacterial infection** | |  | **Fungal and bacterial infection** | |  | **No infection** | |  |
| --- | --- | --- | --- | --- | --- | --- | --- | --- | --- |
|  | SAA | VSAA | P | SAA | VSAA | P | SAA | VSAA | P |
| IL-4 | 3.55(1.82-5.17) | 2.36(1.73-5.34) | 0.2783 | 2.82(1.97-7.49) | 3.72(1.69-5.84) | 0.6927 | 2.88(2.01-4.04) | 2.55(0.98-3.35) | 0.1076 |
| IL-5 | 2.39(1.73-3.70) | 2.27(1.70-3.57) | 0.8261 | 2.29(1.62-3.44) | 2.75(1.94-4.73) | 0.1976 | 2.22(1.66-3.32) | 1.93(1.45-2.71) | 0.1274 |
| IL-6 | 23.47(16.36-34.98) | 32.57(21.01-41.13) | 0.0120 | 137.77(87.05-912.45 | 202.55(76.67-550.65) | 0.6551 | 7.95(6.03-10.27) | 9.13(7.02-14.59) | 0.1595 |
| IL-8 | 25.6(14.65-57.06) | 35.07(11.18-66.46) | 0.7599 | 147.77(43.11-275.25) | 113.21(82.86-448.20) | 0.4497 | 12.58(6.46-30.69) | 36.31(19.83-96.91) | 0.0004 |
| IL-10 | 5.3(4.22-9.92) | 4.35(3.64-6.86) | 0.1024 | 7.10(5.15-15.44) | 7.69(5.01-16.75) | 0.9315 | 4.42(3.30-6.54 | 4.06(3.37-6.44) | 0.9326 |
| IL-12p70 | 4.61(3.43-6.51) | 3.99(3.58-6.27) | 0.7024 | 6.03(5.01-7.56) | 4.27(3.43-5.16) | 0.0046 | 4.00(3.33-5.83) | 3.89(3.03-5.08) | 0.5260 |
| IL-1β | 1.8(1.57-2.57) | 1.62(1.34-2.27) | 0.0882 | 2.03(1.62-2.74) | 1.93(1.32-2.96) | 0.4706 | 1.64(1.17-2.17) | 1.80(1.16-2.31) | 0.9135 |
| IL-2 | 3.65(2.59-10.84) | 3.32(2.60-4.34) | 0.2179 | 3.90(2.82-4.78) | 5.13(3.47-7.35) | 0.0502 | 3.07(2.28-5.30) | 3.68(1.67-4.80) | 0.8872 |
| IFN-γ | 3.07(2.51-3.98) | 3.34(2.30-5.46) | 0.6535 | 3.44(2.02-4.22) | 4.29(2.79-10.09) | 0.0279 | 2.88(1.91-3.79) | 2.83(1.80-5.31) | 0.7490 |
| TNF-α | 3.11(2.09-4.71) | 3.22(2.03-4.23) | 0.8039 | 3.46(2.17-4.22) | 3.78(2.80-5.45) | 0.1306 | 2.88(2.11-4.00 | 2.59(2.03-3.39) | 0.2343 |
| TNF-β | 3.22(2.66-4.01) | 3.09(2.58-3.64) | 0.2057 | 3.69(2.95-4.50) | 3.37(2.81-4.40) | 0.6183 | 3.12(2.62-3.87) | 3.55(2.74-4.28) | 0.2614 |
| IL-17A | 2.8(1.73-4.32) | 2.4(1.62-4.32) | 0.8448 | 2.75(1.93-4.73) | 3.35(2.50-4.73) | 0.3903 | 2.37(1.43-3.36) | 1.68(1.19-2.13) | 0.0729 |
| IL-17F | 4.2(3.07-6.58) | 5.31(3.48-7.16) | 0.3493 | 4.12(2.53-5.81) | 4.02(2.96-5.33) | 0.9863 | 3.36(2.47-4.68) | 3.15(2.74-4.74) | 0.8657 |
| IL-22 | 1.29(0.64-2.91) | 1.02(0.69-2.71) | 0.8187 | 1.74(1.01-2.17) | 1.51(1.01-2.76) | 0.8568 | 1.01(0.60-1.77) | 0.81(0.36-1.56) | 0.5104 |

The difference in the expression of cytokines between the VSAA and SAA detected by the Mann-Whitney U test. P<0.05 means there is a statistical difference.

Supplementary Table 2. AUC between different infection types in SAA and VSAA patients

|  | Between no infection and bacterial lung infection（SAA） |  | Between no infection with pulmonary bacterial and fungal infection（SAA） |  | Between no infection and bacterial lung infection（VSAA） |  | Between no infection with pulmonary bacterial and fungal infection（VSAA） |  |
| --- | --- | --- | --- | --- | --- | --- | --- | --- |
| parameter | AUC | P | AUC | P | AUC | P | AUC | P |
| IL-6 | 0.91 | 0 | 1 | 0 | 0.933 | 0 | 1 | 0 |
| IL-8 | 0.69 | 0 | 0.87 | 0 | — | — | 0.81 | 0.001 |
| IL-10 | 0.64 | 0.0030 | 0.75 | 0 | — | — | 0.73 | 0.01 |

The AUC obtained from ROC curve. P<0.05 means there is a statistical difference.

**Supplementary Figure 1**


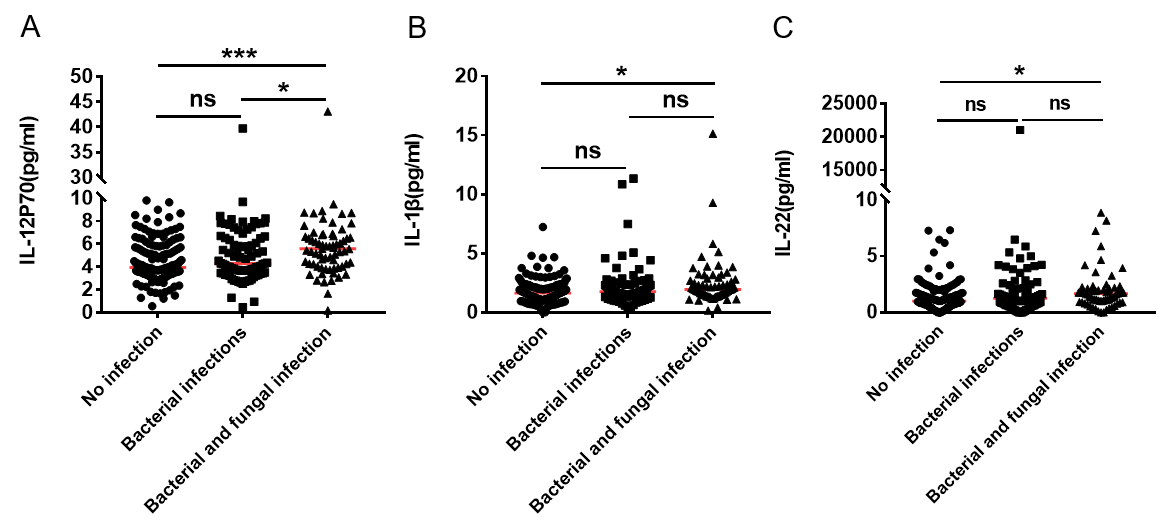


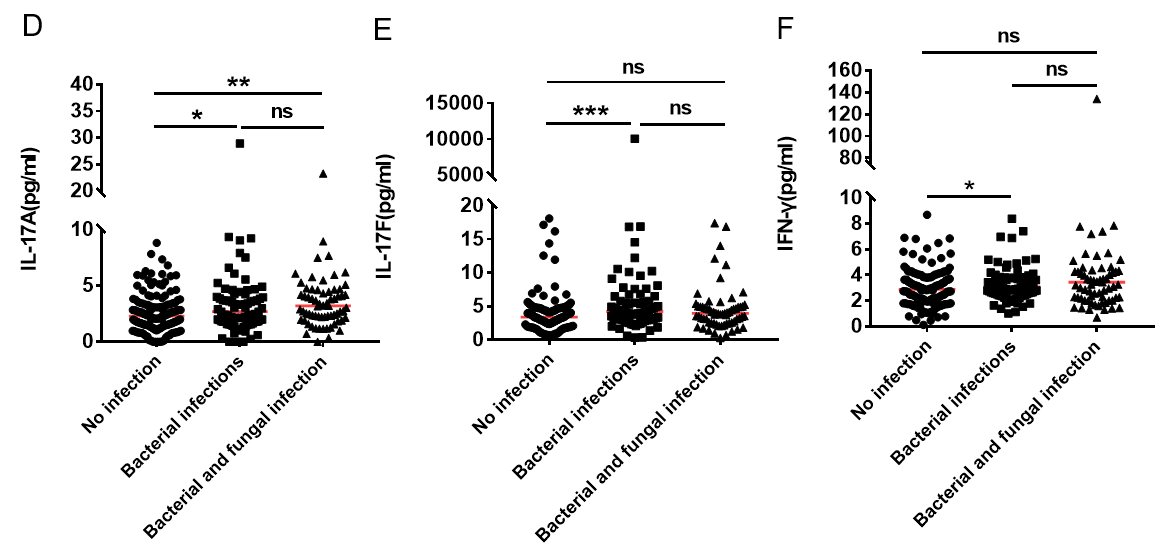


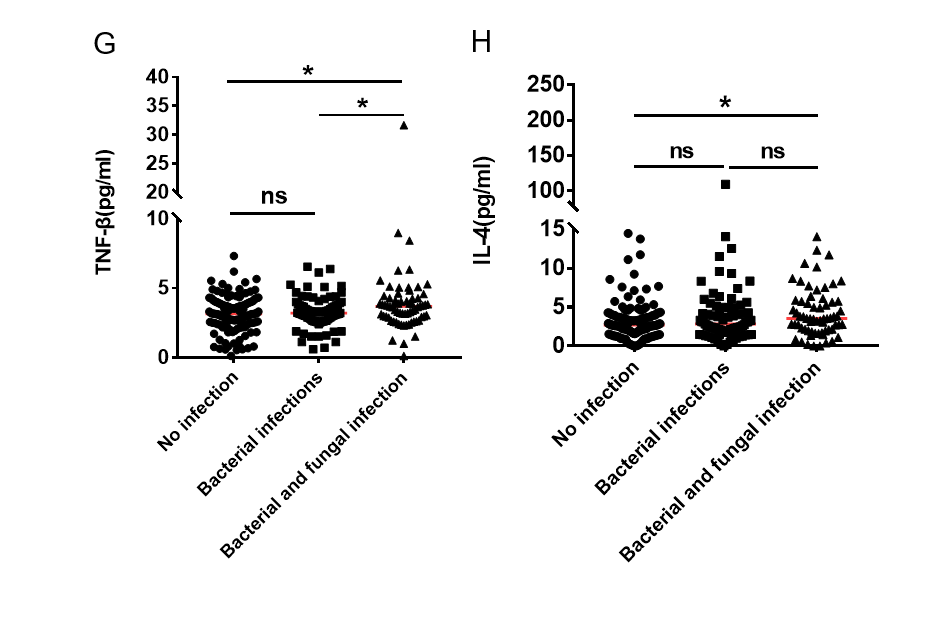


**Supplementary Figure 1**. Differences of IFN-γ, IL-1β, IL-12p70, TNF-α, TNF–β, IL-2, IL-4, IL-5, IL-22, IL-17F and IL-17A among different groups. *P <0.05, **P <0.01, ***P <0.001, ****P < 0.0001, ns: no statistical difference.

**Supplementary Figure 2**


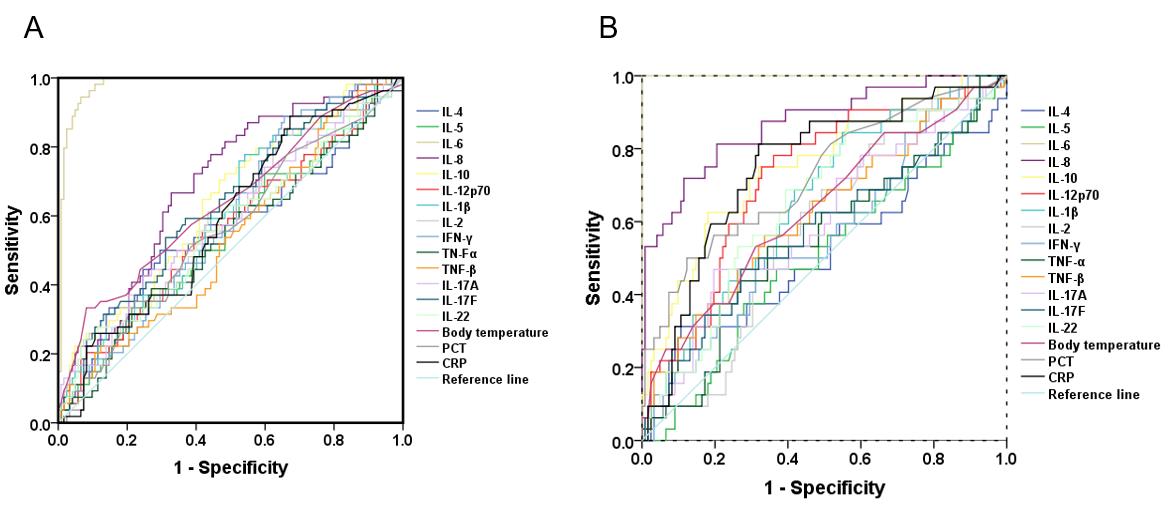


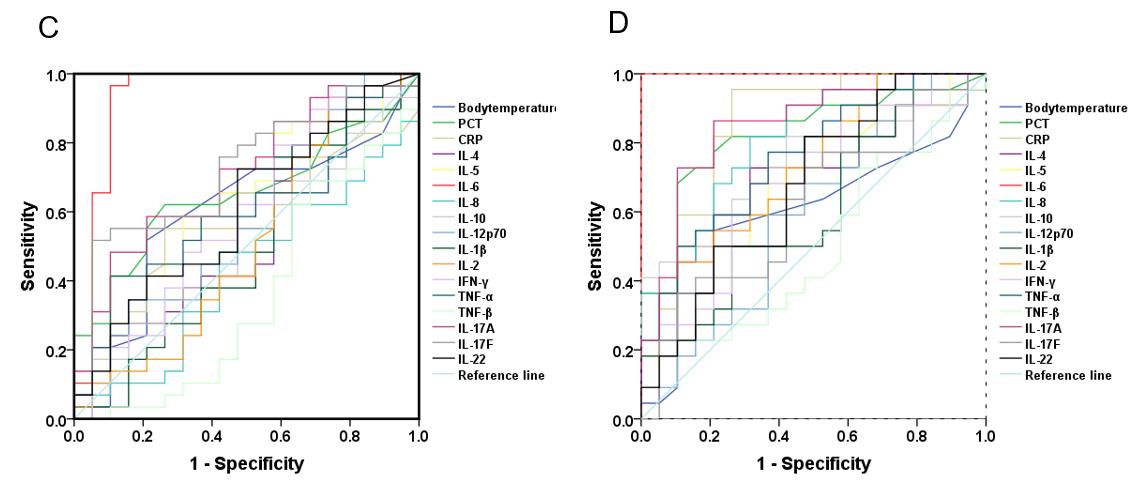


**Supplementary Figure 2.** (A) ROC curve of cytokines, CRP, PCT and body temperature in SAA between patients without infection and patients with pulmonary bacterial infection. (B) ROC curve of cytokines, CRP, PCT and body temperature in SAA between patients without infection and patients with pulmonary bacterial and fungal infection. (C) ROC curve of cytokines, CRP, PCT and body temperature in VSAA between patients without infection and patients with pulmonary bacterial infection. (D) ROC curve of cytokines, CRP, PCT and body temperature in VSAA between patients without infection and patients with pulmonary bacterial and fungal infection.
